# Estimating Hantavirus Risk in Southern Argentina: A GIS-Based Approach Combining Human Cases and Host Distribution

**DOI:** 10.3390/v6010201

**Published:** 2014-01-14

**Authors:** Veronica Andreo, Markus Neteler, Duccio Rocchini, Cecilia Provensal, Silvana Levis, Ximena Porcasi, Annapaola Rizzoli, Mario Lanfri, Marcelo Scavuzzo, Noemi Pini, Delia Enria, Jaime Polop

**Affiliations:** 1 Instituto de Altos Estudios Espaciales “Mario Gulich”, Centro Espacial Teófilo Tabanera, CONAE, Ruta Provincial C45, Km 8, Falda del Carmen, Córdoba 5187, Argentina; E-Mails: ximena.porcasi@conae.gov.ar (X.P.); lamfri@conae.gov.ar (M.L.); scavuzzo@conae.gov.ar (M.S.); 2 GIS and Remote Sensing Unit and Animal Ecology Unit, Department of Biodiversity and Molecular Ecology, Research and Innovation Center, Fondazione Edmund Mach, Via E. Mach 1, San Michele all’Adige, Trento 38010, Italy; E-Mails: markus.neteler@fmach.it (M.N.); duccio.rocchini@fmach.it (D.R.); annapaola.rizzoli@fmach.it (A.R.); 3 Departamento de Ciencias Naturales, Universidad Nacional de Río Cuarto, Ruta 36 Km 601, Agencia Postal N° 3, Río Cuarto, Córdoba 5800, Argentina; E-Mails: cprovensal@exa.unrc.edu.ar (C.P.); jpolop@exa.unrc.edu.ar (J.P.); 4 Instituto Nacional de Enfermedades Virales Humanas “Dr. Julio I. Maiztegui” (INEVH), Monteagudo 2510, Pergamino, Buenos Aires 2700, Argentina; E-Mails: slevis0@yahoo.com (S.L.); pininoemi@yahoo.com.ar (N.P.); deliaenria@gmail.com (D.E.)

**Keywords:** Argentina, *Oligoryzomys longicaudatus*, Andes virus (ANDV), hantavirus pulmonary syndrome (HPS), Species Distribution Models (SDM), Geographic Information Systems (GIS), risk

## Abstract

We use a Species Distribution Modeling (SDM) approach along with Geographic Information Systems (GIS) techniques to examine the potential distribution of hantavirus pulmonary syndrome (HPS) caused by Andes virus (ANDV) in southern Argentina and, more precisely, define and estimate the area with the highest infection probability for humans, through the combination with the distribution map for the competent rodent host (*Oligoryzomys longicaudatus*). Sites with confirmed cases of HPS in the period 1995–2009 were mostly concentrated in a narrow strip (~90 km × 900 km) along the Andes range from northern Neuquén to central Chubut province. This area is characterized by high mean annual precipitation (~1,000 mm on average), but dry summers (less than 100 mm), very low percentages of bare soil (~10% on average) and low temperatures in the coldest month (minimum average temperature −1.5 °C), as compared to the HPS-free areas, features that coincide with sub-Antarctic forests and shrublands (especially those dominated by the invasive plant *Rosa rubiginosa*), where rodent host abundances and ANDV prevalences are known to be the highest. Through the combination of predictive distribution maps of the reservoir host and disease cases, we found that the area with the highest probability for HPS to occur overlaps only 28% with the most suitable habitat for *O. longicaudatus*. With this approach, we made a step forward in the understanding of the risk factors that need to be considered in the forecasting and mapping of risk at the regional/national scale. We propose the implementation and use of thematic maps, such as the one built here, as a basic tool allowing public health authorities to focus surveillance efforts and normally scarce resources for prevention and control actions in vast areas like southern Argentina.

## 1. Introduction

Hantavirus pulmonary syndrome (HPS), an acute respiratory illness fatal in 10%–50% of cases [1], is a severe disease caused by viruses of the Bunyaviridae family. These viruses are zoonotic, host-specific RNA-viruses that persistently infect murid or cricetid rodents of the subfamilies, Murinae, Arvicolinae, Neotominae and Sigmodontinae [2]. Hantaviruses are also known to be carried by shrews, moles and bats [3,4,5]. To date, at least 43 hantavirus genotypes have been described, and about half of them are known to cause HPS in humans [2,6]. Each hantavirus is usually hosted by a single host species in which it establishes a chronic, asymptomatic infection that involves the shedding of infectious virus into the environment in host urine, feces and saliva. These characteristics are key to the transmission of the virus to humans and among rodents [7,8,9,10].

Researchers have long acknowledged that the dynamics of a host population and its relationship with the environmental conditions determine the extent to which a pathogen may persist or disappear, therefore affecting the transmission risk for humans [11,12]. If the chain of relationships, known as the *trophic cascade* hypothesis, holds true [13,14], we would be able to predict transmission risk to humans from climatic and environmental features of sites with confirmed cases of a certain zoonotic disease [12]. The trophic cascade model was originally proposed to explain the number of plague and HPS human cases in the southwestern USA [13,14]. The authors hypothesized that high precipitation (mediated by El Niño phenomenon) increases plant productivity, which, in turn, increases the rodent density. Higher rodent densities lead to higher contact rates, a higher probability of contact with humans and a higher probability of transmission. This model was originally thought of for temporal dynamics, but if we think of it as taking place in the spatial dimension, we may assume that all these relationships occur in every point (or cell) of the space and, consequently, determine the distribution and abundance of hosts, pathogens and human cases of the disease.

Vector-borne and zoonotic diseases display clear spatial patterns that relate to different space-dependent factors: (a) the spatial distribution of vectors and reservoir hosts; (b) the pathogen dispersal ability (conditioned by host or vector dispersal, landscape configuration, *etc*.) and; (c) the human exposure to the infectious agent [15]. In that context, Geographical Information Systems (GIS) and remote sensing (RS) may represent proper tools to describe the spatial distribution of infectious diseases and predict disease risk. These tools have already been used to explain or predict tick-borne [16] and rodent-borne diseases [17,18,19], relating spatial data on land cover and climate to the ecology of the vector or hosts. Species distribution models (SDM) help to delineate the specific habitat requirements of a species [20,21]; coupled with GIS and RS tools, these models can be extrapolated to produce maps displaying the spatial configuration of suitable habitats [22]. These maps are a basic tool for many aspects of resource management and conservation planning [22,23].

How does a trophic cascade relate to SDM? In our view, the trophic cascade model described above may be considered a movie, a temporal film; and SDM for host, pathogen and disease cases as screen-shots of that movie, instantaneous pictures that are the result of environment-host-human interactions. Therefore, if we assume that the number of human cases is related to the density of host (higher density, higher probability of virus transmission among hosts and from hosts to humans) and that this latter issue is determined by physical and biological environmental conditions and we model the relationship between hosts and environment or directly between human cases and the environment, we can predict and map those areas with a higher probability of disease occurrence [12].

In Argentina, four distinct HPS-endemic areas have been recognized [24,25,26]: Northwest (Salta, Jujuy and Formosa provinces), Northeast (Misiones Province), Central (Buenos Aires, Santa Fe and Entre Ríos provinces) and Southern (Neuquén, Río Negro, Chubut and Santa Cruz provinces). These regions differ in their landscape types, vegetation types, agricultural production and land management practices. Hantavirus strains and reservoir hosts differ among endemic areas, and HPS cases are unevenly distributed in space and time in the four regions. The mouse, *Oligoryzomys longicaudatus* (Bennet, 1832), commonly known as colilargo, is the reservoir of Andes virus (ANDV), the hantavirus responsible for HPS in southern Argentina and Chile [27,28]. The colilargo is a widespread rodent highly abundant in the woods and shrublands in Chile and southwestern Argentina [19,29,30,31,32,33,34]. However, it has also been captured in the steppe and in disturbed habitats, such as the borders of cultivated fields, peridomestic settings and pastures [32,33]. The species presents seasonal changes in abundances [33] and irregular outbreaks (“ratadas”) that have been related to bamboo blooming and masting events [35,36]. Besides, the population dynamics of colilargos has been related to precipitations and global climatic indexes [37,38]. Antibody prevalence rates in *O. longicaudatus* show spatial and temporal variations, reaching values of almost 50% in some springs [10,31,32,33,39]. During disease outbreaks, lethality has reached levels of ~50% [40]. In addition to its virulence, ANDV epidemiology is slightly complicated by occasional person-to-person transmission, a feature unique to this particular hantavirus strain [41,42,43]. These aspects make the understanding of the system as a whole an issue of particular relevance for public health.

The relationship of the environment to HPS occurrence is a recent topic of study in Argentina. Hence, if ecological factors affect the distribution of HPS and these factors can be identified, models can be developed to predict the potential distribution of yet unknown foci. Therefore, given the lethality of the disease and the possibility of human-to-human transmission in southern Argentina, it is of great interest to identify and explain the environmental variables associated with HPS case occurrence in order to recognize and predict those places where the risk is higher, allowing public health authorities to focus surveillance efforts and concentrate resources where the need is greatest. Previous works have already assessed the distribution of *O. longicaudatus* in Argentina [19,44,45]. Here, we model and map the distribution of HPS cases caused by ANDV in southern Argentina, to determine the relationship between case occurrence and environmental variables and, more precisely, define and estimate the area with the highest infection probability for humans, through the combination with the distribution map for the rodent host [19].

## 2. Results

From 1995 to 2009, a total of 149 HPS cases were confirmed in southern Argentina as being caused by ANDV. The great majority of them were concentrated in the Andean region of Neuquén, Río Negro and Chubut provinces (40, 54 and 54, respectively). The cases occurred mostly in forest habitats (between 30% and 60%, according to the classification scheme considered) with shrublands in second place (15%–25%, according to the classification considered). The only case registered in Santa Cruz province occurred in a steppe habitat and constitutes the southernmost confirmed case in Argentina (occurring >800 km from the core endemic area of Patagonia).

**Figure 1:**
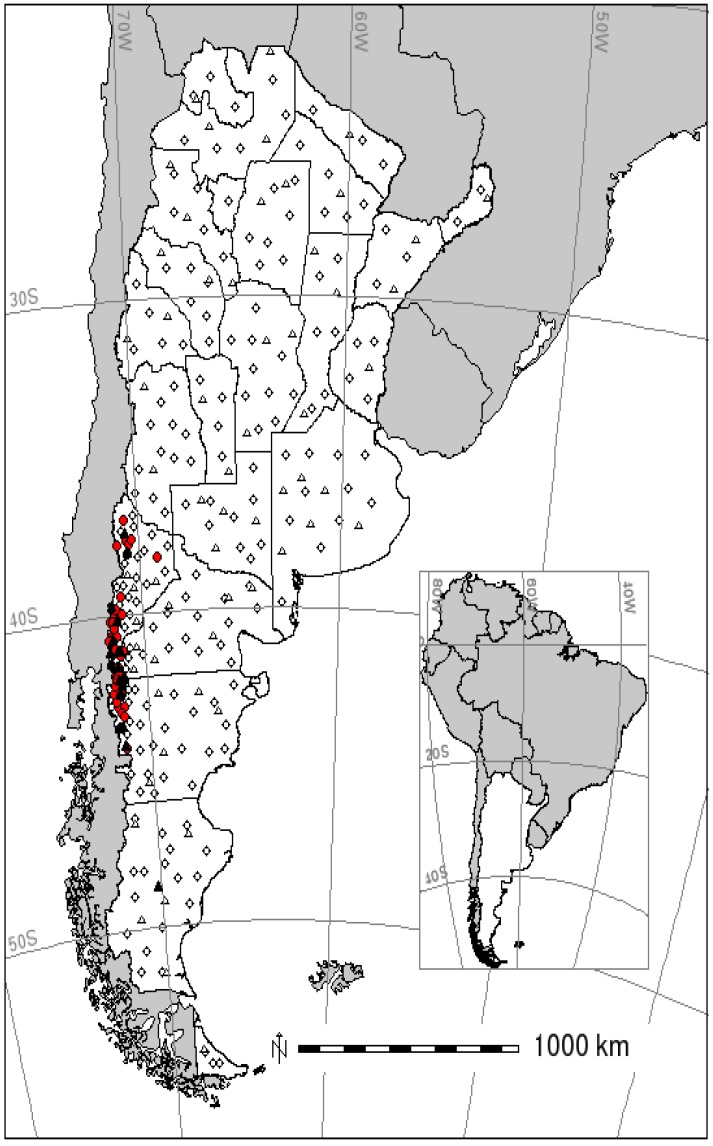
Training and test samples for the occurrence of hantavirus pulmonary syndrome (HPS) in Argentina. Training data in circles; test data in triangles. Filled symbols are the presence and empty symbols the absence of occurrence.

Sites with and without HPS cases by ANDV (Figure 1) differ significantly for most of the environmental variables considered (Table 1). Most precipitation related variables showed a positive association with HPS occurrence, while temperature related ones showed a negative association with case distribution (Table 1).

The best multivariate binomial generalized linear models (GLM) in terms of Akaike’s information criterion (AIC) (m1 and m2, Table 2), included the percent of bare soil (bare), isothermality (bio3), minimum temperature of the coldest month (bio6), mean annual precipitation (bio12) and precipitation of the warmest and coldest seasons (bio18 and bio19, respectively). Variables bare, bio3 and bio18 were negatively associated with the probability of HPS, while bio6, bio12 and bio19 showed a positive relationship. The model with the lowest AIC (m1) was spatialized and selected to draw inferences. In the Maximum Entropy (MaxEnt) model (run with the same predictors as m1), environmental variables showed the same general pattern of association with HPS occurrence, although bio6, bio12 and bio18 presented non-lineal relationships.

**Figure 2:**
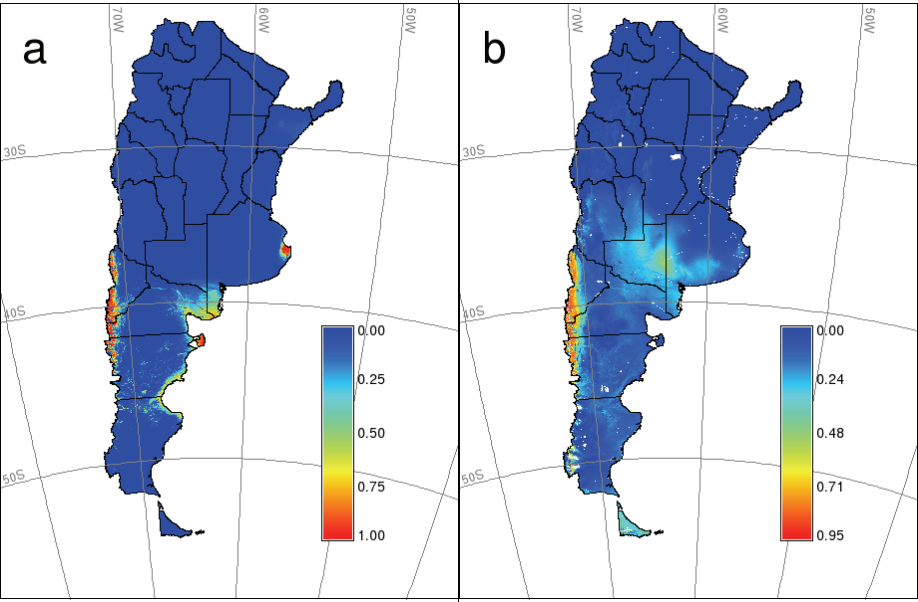
Predicted potential geographic distribution of HPS caused by Andes virus (ANDV) in southern Argentina. (**a**) binomial generalized linear model; (**b**) Maximum Entropy (MaxEnt) model.

Distribution maps (GLM and MaxEnt) for HPS in southern Argentina (Figure 2) showed a high probability area in a narrow strip along the Andes range (approximately 90 km in the widest part and almost 900 km long) from the northwest of Neuquén to the center of Chubut province. The GLM predictive map shows two other “high probability” areas, where no cases by ANDV have ever been reported: Valdés peninsula (Chubut) and Samborombón bay (Buenos Aires). Besides, it shows moderate or moderate-high probability areas in some other places of the Atlantic coast (southern Buenos Aires, north-central Río Negro, south-central Chubut and northern Santa Cruz), where, to the best of our knowledge, no cases have been ever declared. On the other hand, the predictive map obtained with MaxEnt extends (discontinuously) the moderate-high probability area until the southwest of Santa Cruz and Tierra del Fuego provinces along the Andes range. The great majority of confirmed cases occurred in the high probability area in both predictive maps, except for a few of them in the north and east of Neuquén province and the only case of Santa Cruz province (Figure 2).

**Table 1:** Univariate statistics for the environmental variables considered in sites with and without HPS cases by Andes virus in southern Argentina.

Variable	Description and units	HPS	Mean	SD	Median	K	B
ALT	Elevation above sea level (m)	0	739.39	825.78	518.50	4,364.5 ***	0.000018 ns
		1	749.03	358.73	726.00		
BARE	Percentage of bare soil cover (%)	0	34.26	33.94	26.00	8,506 ***	−0.0447 ***
		1	7.51	14.46	0.00		
HERB	Percentage of grass cover (%)	0	56.13	29.12	61.00	5,991.5 ns	−0.0024 ns
		1	54.28	23.18	59.00		
TREE	Percentage of tree cover (%)	0	9.61	15.65	3.00	1,909.5 ***	0.05215 ***
		1	38.21	28.50	32.00		
BIO1	Annual mean temperature (x * 10, °C)	0	131.02	53.56	135.50	8,256 ***	−0.0217 ***
		1	89.00	15.34	89.00		
BIO2	Mean diurnal range (Mean month (max-min), in °C)	0	135.33	19.65	136.50	7,854 ***	−0.0308 ***
		1	125.31	12.61	123.00		
BIO3	Isothermality ((BIO2/BIO7) * 100)	0	49.91	3.82	49.00	3,203.5 ***	0.13419 **
		1	51.66	1.87	52.00		
BIO4	Temperature seasonality (SD * 100)	0	4,817.7	691.50	4,823.0	8,748 ***	−0.0015 ***
		1	4,284.7	317.77	4,248.0		
BIO5	Maximum temp of the warmest month (x * 10, °C)	0	273.58	60.68	296.00	8,334.5 ***	−0.0153 ***
		1	228.61	23.10	224.00		
BIO6	Minimum temp of the coldest month (x * 10, °C)	0	5.10	43.74	1.50	6,935.5 ***	−0.0124 **
		1	−11.46	10.41	-11.00		
BIO7	Temperature annual range (BIO5-BIO6) (x * 10, °C)	0	268.48	35.24	270.00	8,749 ***	−0.0302 ***
		1	240.07	18.52	237.00		
BIO8	Mean temp of the wettest quarter (x * 10, °C)	0	141.24	95.40	152.00	8,886.5 ***	−0.0207 ***
		1	39.57	15.67	41.00		
BIO9	Mean temp of the driest quarter (x * 10, °C)	0	112.47	39.82	109.50	2,965 ***	0.0273 ***
		1	143.26	18.64	142.00		
BIO10	Mean temp of the warmest quarter (x * 10, °C)	0	191.63	56.51	203.00	8,519.5 ***	−0.0201 ***
		1	144.44	18.17	143.00		
BIO11	Mean temp of the coldest quarter (x * 10, °C)	0	68.54	51.69	69.00	7,706.5 ***	−0.0201 ***
		1	34.49	12.78	35.00		
BIO12	Annual precipitation (mm)	0	494.92	338.62	402.50	1,705 ***	0.00386 ***
		1	976.97	309.86	1,011.0		
BIO13	Precipitation of the wettest month (mm)	0	75.13	48.68	63.50	1,182.5 ***	0.0351 ***
		1	168.69	52.52	170.00		
BIO14	Precipitation of the driest month (mm)	0	15.34	14.54	11.00	1,835.5 ***	0.0623 ***
		1	28.67	11.41	26.00		
BIO15	Precipitation seasonality (variation coefficient)	0	48.94	23.73	45.50	3,915 ***	0.0200 **
		1	58.02	7.70	58.00		
BIO16	Precipitation of the wettest quarter (mm)	0	201.12	131.03	177.50	1,081 ***	0.01396 ***
		1	455.36	131.42	476.00		
BIO17	Precipitation of the driest quarter (mm)	0	54.09	49.44	40.00	1,728.5 ***	0.02017 ***
		1	105.52	40.98	101.00		
BIO18	Precipitation of the warmest quarter (mm)	0	151.59	128.00	88.00	5,584 ns	−0.00410 **
		1	105.84	41.44	101.00		
BIO19	Precipitation of the coldest quarter (mm)	0	94.67	98.46	56.00	438 ***	0.0159 ***
		1	431.13	129.22	451.00		

BARE: % of bare soil cover; HERB: % of grass cover; TREE: % of woody cover; HPS (0): HPS absence; HPS (1): HPS presence; SD: standard deviation; K: Kruskal-Wallis chi-squared statistic; B: univariate binomial Generalized Linear Model parameter (parameter significance according to a *t*-test on parameter SD with 245 degrees of freedom); *** *p* < 0.001; ** *p* < 0.01; * *p* < 0.05; *p* < 0.1; ns: not significant, *p >* 0.1.

**Table 2:** Multivariate binomial GLM models for HPS by Andes virus occurrence in southern Argentina.

Model	Variables	AIC	ΔAIC
m1	BARE + BIO3 + BIO6 + BIO18 + BIO12	76.07	0.00
m2	BARE + BIO3 + BIO6 + BIO18 + BIO19	76.76	0.69
m3	BARE + BIO3 + BIO6 + BIO18 + BIO19 + TREE	78.32	2.25
m4	BIO3 + BIO6 + BIO12 + BIO18	79.79	3.71
m5	BARE + BIO3 + BIO4 + BIO6 + BIO18 + BIO19 + TREE	79.86	3.78
m6	BIO3 + BIO4 + BIO6 + BIO12 + BIO18	80.69	4.62
m7	BIO3 + BIO6 + BIO18 + BIO19 + TREE	80.71	4.63
m8	HERB + BIO3 + BIO6 + BIO18 + BIO12	81.45	5.38
m9	BARE + BIO3 + BIO4 + BIO6 + BIO15 + BIO18 + BIO19 + TREE	81.86	5.78
m10	BIO9 + BIO19	88.12	12.04
m11	BIO12 + BIO19	98.20	22.13
m12	BIO4 + BIO19	100.42	24.35

BARE: % of bare soil cover; HERB: % of grass cover; TREE: % of woody cover; BIO1: Mean annual temperature; BIO3: Isothermality; BIO4: Temperature seasonality; BIO6: Minimum temperature of the coldest month; BIO9: Mean temperature of the dry season; BIO12: Mean annual precipitation; BIO15: Precipitation seasonality; BIO18: Precipitation of the warmest quarter; BIO19: Precipitation of the coldest quarter; AIC: Akaike’s information criterion value; ΔAIC: difference between each model AIC and the one of the lowest AIC.

**Figure 3:**
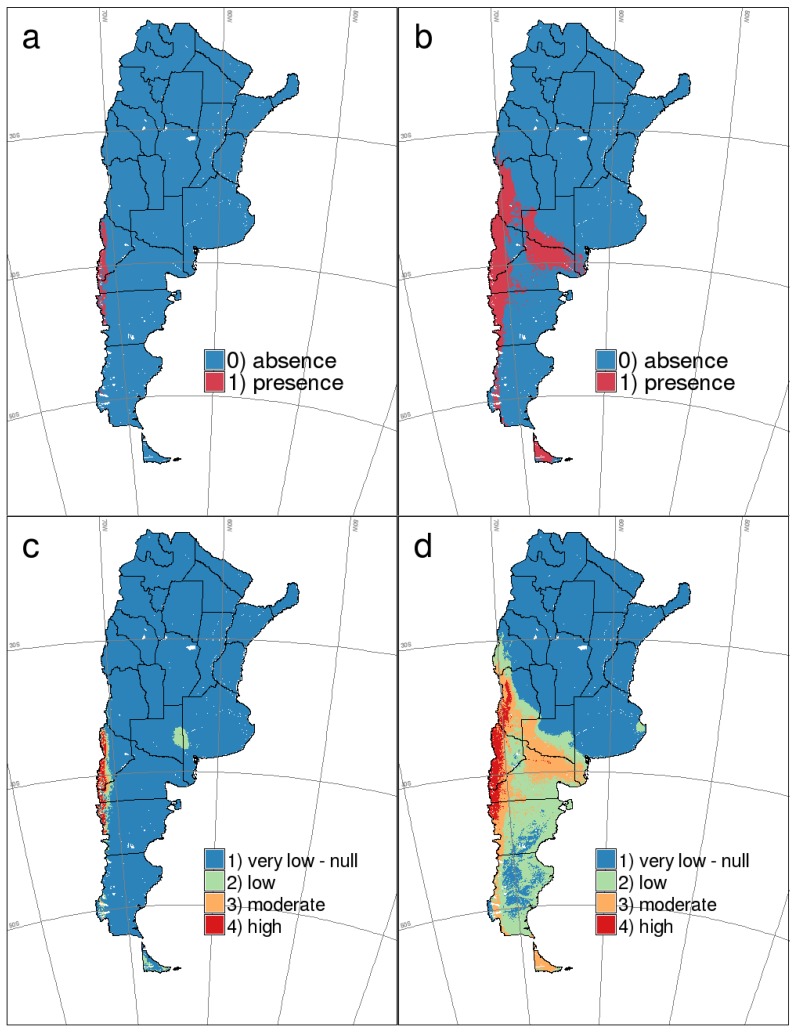
MaxEnt binary (**above**) and reclassified (**below**) maps for HPS cases by ANDV and *O. longicaudatus* presence in southern Argentina. (**a**,**c**) HPS by ANDV; (**b**,**d**) *O. longicaudatus*.

According to receiver operating characteristic (ROC) curves and area under the curve (AUC) values, both models had highly satisfactory performances (0.986 and 0.984, for GLM and MaxEnt models, respectively). However, in the threshold-dependent approach (Table 3), differences in predictive performance between models were more noticeable, especially when considering minimum occurrence prediction as the threshold. These differences were then translated to presence-absence maps (not shown). After analyzing accuracy and error measures for different threshold-selection criteria, the best cut-off probabilities were 0.43 and 0.65 for GLM and MaxEnt models, respectively. As the threshold 0.65 for the MaxEnt model showed the best performance in key indexes, like sensitivity and false negative rate (Table 3), we used this predictive map for further analysis. The binary map built using this threshold (0.65) is shown in Figure 3a. The same analysis was carried out for the host predictive model (not shown), and the resulting binary map is shown in Figure 3b (cut-off probability for MaxEnt model = 0.52). To complement the assessment of the HPS model’s predictive performance, we used the threshold-independent ROC curve and AUC on test samples. Again, models behaved nearly equally: AUC of 0.986 and 0.975, for GLM and MaxEnt models, respectively.

**Table 3:** Comparison of the models’ performance using different criteria for threshold selection. All criteria in the last line for generalized linear model (GLM) and maximum entropy algorithm (MaxEnt) predictions yielded the same cut-off probability. Min, minimum; Sens, sensitivity; Specif, specificity; Max, maximum; Max prop correct, maximum proportion of presence and absence records correctly identified; K, Kappa index; ROC, receiver operating characteristic.

Criteria	Threshold	Sensitivity	Specificity	False positive rate	False negative rate	Positive predictive value	Negative predictive value	K
**GLM**								
Min occurrence prediction	0.019	1.000	0.742	0.258	0.000	0.560	1.00	0.59
Mean occurrence prediction	0.869	0.754	0.989	0.011	0.246	0.958	0.925	0.80
10% omission	0.550	0.902	0.962	0.038	0.098	0.887	0.968	0.86
Sens = Specif, Max Sens + Specif, Max prop correct, Max K, Min ROC plot distance	**0.430**	**0.951**	**0.952**	**0.048**	**0.049**	**0.866**	**0.983**	**0.87**
**MaxEnt**								
Min occurrence prediction	0.093	1.000	0.505	0.495	0.000	0.399	1.000	0.33
Mean occurrence prediction	0.799	0.738	0.989	0.011	0.262	0.957	0.920	0.79
10% omission	0.730	0.902	0.968	0.032	0.098	0.902	0.968	0.87
Sens = Specif	0.570	0.951	0.952	0.048	0.049	0.866	0.983	0.87
Max Sens+Specif, Max prop correct, Max K, Min ROC plot distance	**0.650**	**0.951**	**0.968**	**0.032**	**0.049**	**0.906**	**0.984**	**0.90**

Figure 3c,d show the reclassified maps for HPS and rodent host distribution that were built considering the best thresholds estimated in each case and the analysis of the distribution of predicted probabilities for the presence and absence points in the training datasets. The resulting reclassification rules for mice and HPS were: (1) for mice occurrence: 0.0 − 0.3 = 1; 0.3 − 0.5 = 2; 0.5 − 0.7 = 3 and 0.7 − 1.0 = 4; and (2) for HPS occurrence: 0.0 − 0.35 = 1; 0.35 − 0.6 = 2; 0.6 − 0.75 = 3; 0.75 − 1.0 = 4. The value (1) represents very low or null risk; (2) low risk; (3) moderate risk and (4) high risk.

Overall, the area that resulted in being classified as HPS-positive according to the 0.65 threshold covers a surface of almost 38,000 km^2^, while the predicted area for the host presence (0.52 threshold) encompasses approximately 353,000 km^2^. Therefore, according to the estimated presence thresholds, the actual distribution of the disease comprises an ~11% of the distribution of the reservoir host. When we consider the reclassified maps, the area with the highest probability of HPS occurrence covers approximately 23,500 km^2^; 28% of the area where the probability of finding the rodent host is the highest (Figure 3c,d).

**Figure 4:**
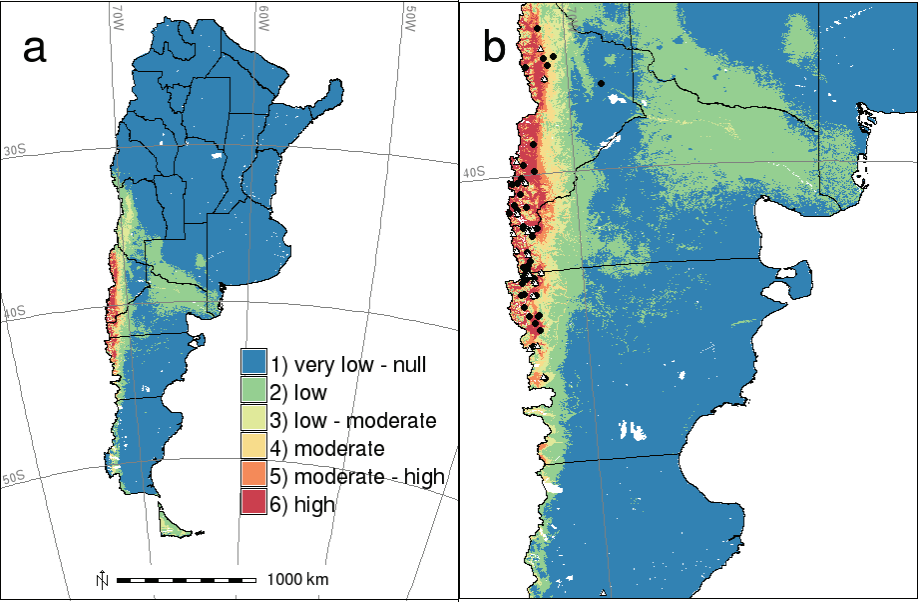
(**a**) Risk map for HPS caused by Andes virus in southern Argentina; (**b**) zoomed map of Patagonia covering HPS occurrence. Training (black circles) and test (white triangles) HPS presence records.

The risk map resulting from combining reclassified maps for host and HPS case distributions is shown in Figure 4. Risk, as well as occurrence probabilities for the host and the disease decreases more or less generally from west to east and from north to south. The highest risk area (class 6), the one combining a high probability of both mice and human disease case occurrences and where most HPS confirmed cases occurred, covers approximately 22,000 km^2^ (700 km in length and 70 km in width). In terms of the type of habitat, this area corresponds to sub-Antarctic *Nothofagus* and *Austrocedrus* forests and shrublands. There are some other disjunctive patches classified as high and moderate-high risk in southwestern Chubut and northwestern Santa Cruz provinces, also characterized by this type of habitats. Then, there is a low risk area along Colorado river, where no HPS cases have been confirmed; but, it has a moderate probability of *O. longicaudatus* occurrence, and only two cases were recorded in areas classified as very low risk (Figure 4b), areas that are steppe habitats.

## 3. Discussion

Sites with confirmed cases of HPS (caused by ANDV) in Patagonia were mostly concentrated in the area with the highest probability of occurrence; a very narrow strip along the Andes range from northern Neuquén to central Chubut province. The highest infection probabilities for humans appeared, then, to be concentrated in this narrow area of ~23,500 km^2^ in the Andean region of sub-Antarctic forests. In general, sites with a higher probability of HPS occurrence in southern Argentina were characterized by high annual mean precipitation (~1,000 mm on average, ranging from 500 in the eastern part to 2,000 mm in the west), but dry summers (from 40 to 250 mm), very low percentages of bare soil (10% on average) and low temperatures in the coldest month (from −3.6 to 0.8 °C). Habitats with these environmental features coincide mostly with sub-Antarctic forests dominated by *Nothofagus* species and *Austrocedrus chilensis* and shrublands, where colilargo abundance, as well as ANDV prevalence are known to be the highest [31,32,33,34,39]. Therefore, the highest abundances of hosts or the highest probabilities of occurrence (which would allow virus persistence and transmission given the assumed higher connectivity among populations) would be good indicators of the highest levels of transmission risk.

Although both models (GLM and MaxEnt) were highly satisfactory in terms of AUC, the output of the MaxEnt model was more precise and showed better performance in the accuracy and error measures considered. Two other reasons for choosing MaxEnt included: (1) it extends the moderate-high probability of occurrence of HPS until southern Santa Cruz and Tierra del Fuego, consistent with previous records [45,46]; and (2) it correctly predicted low occurrence probabilities in areas with no records for ANDV.

The models fitted here assume a static distribution of the host species and disease cases (*i.e.*, in equilibrium with the environment), an assumption usually permitted for modeling purposes. Previous studies, however, have pointed out a tendency towards lower precipitation and higher temperatures for southern South America [47,48]. Although we accept that there may be limitations in using climate data averaged over a 50-year period, it has been shown that most of colilargos’ actual distribution would remain unchanged unless climatic tendencies were double those observed [49]. We may assume, therefore, that our models and maps constitute a good representation of current and future disease distribution.

The emergence of human diseases has often been found to be more spatially restricted than the distribution of the reservoir host [50,51]. This seems to be the case for HPS in southern Argentina, too. In fact, the area with the highest probability for HPS occurrence represents 28% of the highest probability area in the *O. longicaudatus* distribution map (Figure 3). This particular area has a quite low population density (4.5 inhabitants per km^2^ on average; from 0.8 to 24.7; Instituto Nacional de Estadística y Censos, Argentina) [52] and it is characterized by cities of mean urban development (mainly touristic cities), little towns with undefined boundaries and a rural population. The economic activity is mostly related to tourism, crafts and fruit production [24].

Other factors have to be searched regarding the variation of human susceptibility or exposure. In fact, it has been observed that the disease is more frequent in young (21–30 years old) active males [26]. Aside from contact with previous HPS case-patients, it was reported that the most frequent exposure factors for HPS in Patagonia were related to rural work (general work on farms, preparing land for cultivation, clearing weeds, planting and harvesting and cleaning out barns or other outbuildings) and activities in natural environments (recreational activities or tourism) [26]. There is a particular activity that poses quite a high risk of infection: from March to May, local inhabitants collect sweet briar fruits for jam making and the cosmetic industry. Shrublands dominated by this plant have shown the highest abundances of colilargos, and infection was more frequently detected in this kind of habitat [33,34]. In addition, the fruits of sweet briar are one of the most common food items of colilargos year-round [53]. On the other side, variation in the hazard, represented by the infection prevalence of the rodent host, may be due to: (1) the requirement for threshold population densities to sustain infection in the reservoir [54,55]; (2) differences in virus pathogenicity and/or host immunity; (3) the existence of unrecognized cryptic host species that might not support infection [11]; or (4) demographic differences in the human population.

Infected *O. longicaudatus* have been captured along the Andes range in Neuquén, Río Negro and Chubut provinces in Argentina [31,32,33,34,39]. Most cases seem to occur in forest or shrubland habitats (especially those highly covered with the invasive *Rosa rubiginosa*), where the highest host abundance and virus prevalence have been recorded [31,32,33,34,39]. Besides, infected mice have also been captured in shrubby peridomestic settings [33] and sylvan areas of steppe in Chubut province [34]. In fact, the southernmost HPS case recorded in Argentina (48°46'1.2''S; 70°15'0.0''W) occurred in a steppe-like habitat [56]. If we take this into account, the steppe areas that are close to forests and sum up the moderate and high HPS risk classes (classes 4, moderate, 5, moderate-high, and 6, high risk), the total risk area in southern Argentina adds up to 58,000 km^2^ (more than double the high risk area). The population under risk in this area is about 300,000 people (2010 population census, Instituto Nacional de Estadística y Censos, Argentina) [52], a number that increases considerably in the summer months, due to tourism (one of the main economic activities of the region) [24].

In this sense, we understand that the delineation of high risk areas is always relevant, but we also sustain that monitoring should be carried out in those transition areas of moderate/low probabilities that, in light of dramatic environmental or demographic changes, may shift their risk status. For the case of HPS in Patagonia, these are quite unpopulated areas eastwards of the Andes range (most of the human population is concentrated on the west, where the hazard is higher), but that may not be the case for other regions of Argentina or for other diseases in general, where, in the face of environmental or demographic changes, the risk level may increase. It may be worth including demographic variables into models in case they are correlated or confounded with some significant environmental feature [57] or adjusting the hazard by human population density.

Some variables identified as the best predictors in our models implicitly include a seasonal dimension that it is not explicitly considered in the response variable. However, this is where, to our understanding, the temporal aspect of the trophic cascade translates into the spatial distribution of the disease modeled by SDM. Variables retained in models for HPS human cases are consistent with factors affecting the temporal dynamics of hosts (*i.e.*, minimum temperature, precipitation of the warmest season) [38] and, consequently, their spatial distribution, which will then influence the occurrence and distribution of the disease. These same variables were found to be significant for host distribution at different spatial scales [19,34,45]. We may infer, therefore, that these variables reflect factors that influence host dynamics and distribution in areas of high transmission and, consequently, determine the number of human cases, as stated in the trophic cascade model. For example, let us consider the precipitation of the warmest season. We know that the abundance of the host is quite low in summer, and infection prevalence high; higher levels of precipitation would imply more primary productivity (food and refuge). This would favor reproductive activity and would translate into higher abundance the following autumn-winter period. Higher abundance would imply higher transmission among rodents and higher probabilities of transmission to humans [12].

Disease transmission systems represent complex interactions among multiple species (e.g., vectors, hosts, pathogens) and different options exist regarding how they should be analyzed and modeled. Traditionally, methodologies for evaluating the geographic risk of disease transmission have focused on the overall distribution of cases as an epi-phenomenon. Though useful as a primary tool, this approach is only able to identify broad general trends and patterns, giving an overall picture of the ecology of the transmission chain [58]. An alternative, however, would be to model each component species in the transmission system and then assemble them into a geographic picture of the transmission system, as we intend to do in the present work. In our view, the combination of predictive distribution maps of the reservoir host and disease cases, which reflect the actual exposure of humans to the virus, represents an improvement and a step forward in the understanding of the risk factors that need to be considered in regional/national-scale risk forecasting and mapping. Therefore, we propose thematic maps, such as the one obtained here, as basic tools, allowing public health authorities to focus surveillance efforts and commonly scarce resources for prevention and control actions in vast areas, like southern Argentina.

## 4. Materials and Methods

### 4.1. Hantavirus Pulmonary Syndrome Data

Time series data on HPS confirmed cases caused by ANDV in southern Argentina was provided by the Health Ministries of Neuquén, Río Negro and Chubut provinces, covering the period 1995–2009. Further data on confirmed cases was obtained from a literature review [55]. Using information regarding potential infection sites and residence localities, latitude and longitude coordinates were assigned to sites with confirmed cases. The set of coordinates were mostly obtained from the National Geographic Institute GIS database (Instituto Geográfico Nacional, Argentina) [59] through searching for the corresponding place/locality name. HPS localities were considered just once when more than one case was recorded at the same site and when secondary transmission was confirmed or suspected. We deleted sites that were less than 3 km apart to avoid auto-correlation issues. Since the estimation of the potential distribution requires absences located farther apart in the geographic and/or environmental space and we only had “presence” data, we randomly selected localities without confirmed cases of HPS (by ANDV) all over the country [60]. We treated these points as real absences, since HPS declaration is mandatory, and we only used confirmed cases (through laboratory antibody tests). The database consisted, then, of 61 different localities with confirmed cases and 186 points without cases, following common approaches [60,61]. A randomly selected subset of locations (20% of presences and absences) was withheld for validation studies. Records of HPS localities were imported into a GIS using the free and open source software, GRASS GIS 7.0 [62,63].

### 4.2. Environmental Data

Data layers for topography, climate and land cover were compiled for Argentina: Altitude and climatic data layers as 19 bioclimatic variables (of ~1 km^2^ of spatial resolution) were drawn from the WorldClim data set [64]. Land cover data was drawn from the Vegetation Continuous Fields collection (VCFMOD44B, collection 3), which contains proportional estimates for vegetative cover types: woody vegetation, herbaceous vegetation and bare ground [65]. The product was aggregated from an initial 0.5 km to 1 km pixel length to match the resolution of the climatic variables by average value resampling in GRASS GIS. Land cover data from 5 different classification schemes derived from MODIS (Moderate Resolution Imaging Spectroradiometer) sensor imagery (MOD12Q1) were used to estimate the proportion of cases occurring in each type of vegetation cover [66].

### 4.3. Spatial and Statistical Modeling

To characterize the distribution of HPS caused by ANDV, two modeling approaches were compared: generalized linear models (GLM) with binomial error [67] and the Maximum Entropy algorithm (MaxEnt) [68,69,70]. We first performed an exploratory analysis comparing environmental variables between sites with and without HPS cases with Kruskal-Wallis tests. We also conducted univariate binomial GLMs to determine the association between HPS occurrence and altitude, climate and land cover. Variables that did not differ between sites with and without HPS or that were not significant in univariate GLMs were not considered for further analysis. The significance of variables inside models was evaluated with *t*-tests. Variance inflation factors (VIFs) and pairwise Pearson correlation coefficients were computed to evaluate collinearity among the independent variables. Variables with VIFs lower than 10 (and/or that yielded an average VIF of 5) were retained. A multimodel inference approach based on Akaike’s information criterion (AIC) was used, and the resulting best model (lowest AIC) was applied in a GIS to extrapolate the predicted likelihood of occurrence across the entire area of concern and to draw inferences. R 3.0.1 [71] and GRASS GIS 7.0 [62,63] were used for modeling and mapping, respectively. A Moran test was applied to the residuals of this model to assess whether the unexplained variation was randomly distributed (Software Passage version 2.0) [72].

The MaxEnt algorithm was applied using the same combination of predictor variables as in the best GLM model. It was run using MaxEnt software, version 3.3.3k [68,69,70], with the SWD format (‘‘samples with data’’), which allows for the inclusion of both presence and absence data. We used a logistic map as output with values ranging from 0 to 1. All other MaxEnt software parameters were maintained at default settings.

As recommended by Vaughan and Ormerod [73], we assessed the predictive performance of GLM and MaxEnt models with both threshold-dependent and -independent measures using the training dataset, to provide both a general assessment of performance and one specific to particular thresholds, and to obtain a more accurate picture of the predictive behavior of the models. Using receiver operating characteristic (ROC) curves, we assessed the overall discrimination ability of each model on the basis of the area under the ROC curve (AUC) as the threshold-independent measure. However, as predictive modeling also requires a threshold probability at which to accept the occurrence of the entity being modeled, we complemented the former evaluation with a threshold-dependent approach, which entails selecting a threshold for converting probabilities of occurrence into binary data [74,75,76]. We compared different criteria for threshold selection [77] using the package, SDMTools 1.1-13 in the R Language and Environment for Statistical Computing [78]. The criteria considered were: minimum occurrence prediction, mean occurrence prediction, 10% omission, sensitivity = specificity, maximum sensitivity + specificity, maximum kappa, maximum proportion of presence and absence records correctly identified and min-ROC (plot distance; the threshold value or range of values where the ROC curve is closest to point 0, 1). For each threshold, we obtained a binary map and confusion matrix that allowed us to depict the modeled spatial distribution and to provide other estimates of model accuracy (Table 4) based on comparing observed *versus* predicted presences and absences [75,79,80,81]. Finally, the test dataset was used to compare the performance of the models using only a threshold-independent measure. ROC and AUC were estimated using the package, ROCR, version 1.0-5 for R [82].

**Table 4:** Threshold-dependent measures used for assessing the predictive performance of models. TP, the number of presence points correctly classified as present; TN, the number of absence points correctly classified as absent; FP, the number of actual absence points classified as present; FN, the number of actual presence points classified as absent; P, the total number of actual presences; N, the total number of actual absences.

Performance measure	Definition	Formula
Sensitivity (True positive rate)	Proportion true presences correctly predicted	TP/P
Specificity (True negative rate)	Proportion true absences correctly predicted	TN/N
False positive rate		FP/N
False negative rate		FN/P
Positive predictive value (Precision)	Percentage of predicted presences that were real	TP/(TP + FP)
Negative predictive value	Percentage of predicted absences that were real	TN/(TN + FN)

### 4.4. HPS Risk Mapping: Integration with Previous Potential Distribution Map of Host

**Figure 5:**
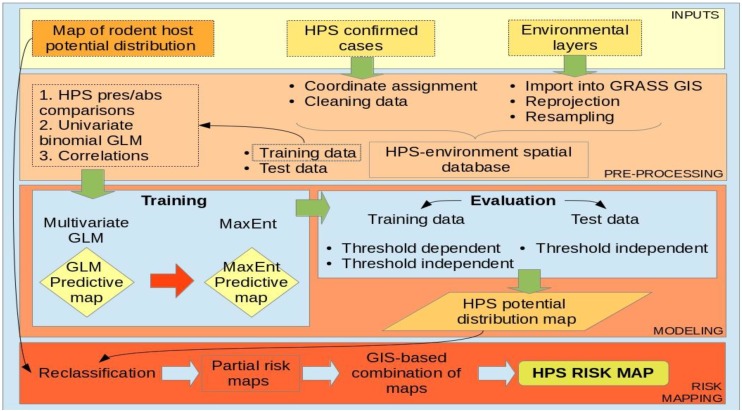
Schematic representation of the methodological workflow.

The existing continuous potential distribution map for rodent host [19] and the HPS distribution map obtained in the present study were reclassified into four intervals reflecting levels of risk (in general, the higher the probability, the higher the risk). For the reclassification, we considered the thresholds estimated previously (as described above) and the analysis of the distribution of predicted probabilities for the presence and absence points in the training datasets of both host and human cases. Maps for reservoir host and HPS were then added up to represent the different categories of risk considering the combination of host and HPS presence/absence and assuming the highest risk to occur, where both *O. longicaudatus* and HPS cases showed the highest probability of presence (Figure 5).

## 5. Conclusions

The present analysis of HPS and *O. longicaudatus* occurrence in southern Argentina contributes to a better understanding of the system and the distribution of transmission risk. Although HPS is a relatively rare disease in Argentina (more than 1,000 cases between 1995 and 2009 over all of the country) [74] and cases provoked by ANDV constitute 16.5% of the cases declared in the country [45], the disease is among the most pathogenic (~50% lethality) of human viral infections. ANDV is the only hantavirus that presents the ability to be transmitted among humans. As more cases are recognized and risk factors are better identified, it will be possible to enhance surveillance efforts and to evaluate prevention measures. We emphasize and highlight that when human-to-human transmission occurs, there is the *need* for improving surveillance.
